# Acetylated α-Tubulin Regulated by N-Acetyl-Seryl-Aspartyl-Lysyl-Proline(Ac-SDKP) Exerts the Anti-fibrotic Effect in Rat Lung Fibrosis Induced by Silica

**DOI:** 10.1038/srep32257

**Published:** 2016-08-31

**Authors:** Wang Xiaojun, Liu Yan, Xu Hong, Zhang Xianghong, Li Shifeng, Xu Dingjie, Gao Xuemin, Zhang Lijuan, Zhang Bonan, Wei Zhongqiu, Wang Ruimin, Darrell Brann, Yang Fang

**Affiliations:** 1 Basic Medical Collage, Hebei Medical University, Shijiazhuang, China; 2 Medical Research Center, North China University of Science and Technology, Tangshan, China; 3 Traditional Chinese Medicine Collage, North China University of Science and Technology, Tangshan, China; 4 Department of Neuroscience and Regenerative Medicine, Medical College of Georgia, Augusta University, Augusta, GA 30912, USA

## Abstract

Silicosis is the most serious occupational disease in China. The objective of this study was to screen various proteins related to mechanisms of the pathogenesis of silicosis underlying the anti-fibrotic effect of N-acetyl-seryl-aspartyl-lysyl-proline (Ac-SDKP) using proteomic profile analysis. We also aimed to explore a potential mechanism of acetylated α-tubulin (α-Ac-Tub) regulation by Ac-SDKP. Two-dimensional electrophoresis (2-DE) and matrix-assisted laser desorption ionization time-of-flight mass spectrometry (MALDI-TOF/TOF MS) were used to assess the different protein expression profiles between control and silicosis rats treated with or without Ac-SDKP. Twenty-nine proteins were identified to be potentially involved in the progression of silicosis and the anti-fibrotic effect of Ac-SDKP. Our current study finds that 1) the lost expression of Ac-Tub-α may be a new mechanism in rat silicosis; 2) treatment of silicotic rats with N-acetyl-Seryl-Aspartyl-Lysyl-Proline (Ac-SDKP) inhibits myofibroblast differentiation and collagen deposition accompanied by stabilizing the expression of α-Ac-Tub *in vivo* and *in vitro*, which is related with deacetylase family member 6 (HDAC6) and α-tubulin acetyl transferase (α-TAT1). Our data suggest that α-Ac-Tub regulation by Ac-SDKP may potentially be a new anti-fibrosis mechanism.

Recently, the naturally occurring tetrapeptide N-acetyl-seryl-aspartyl-lysyl-proline (Ac-SDKP) was recognized as an anti-fibrotic peptide that contributes to the prevention of inflammation and fibrosis in cardiovascular^1^, autoimmune^2^, pulmonary^3^, liver^4^ and renal diseases^5^. At basal levels, endogenous Ac-SDKP can antagonize and prevent excessive collagen deposition. In contrast, chronic blockade of Ac-SDKP promotes and accelerates collagen accumulation in the presence of pro-fibrotic stimuli, such as angiotensin II (Ang II)^6^. It has been reported that tuberculous pericarditis is associated with low levels of Ac-SDKP in normal pericardial fluid^7^. In addition, Ac-SDKP has been shown to inhibit cell proliferation, myofibroblast differentiation, and collagen deposition, effects that are regulated by transforming growth factor beta (TGF-β) signaling and the rennin-angiotensin system (RAS), among other systems^1^,^2^,^3^,^4^,^5^,^6^,^7^. These observations suggest that Ac-SDKP has an anti-fibrotic effect and that fibrotic diseases are mediated, at least in part, by the down-regulation of Ac-SDKP.

In the past few years, our research has focused on the anti-fibrotic effect of Ac-SDKP in a rat model of silicosis. We previously demonstrated that Ac-SDKP could prevent lung fibrosis induced by SiO_2_ *in vivo* and *in vitro*^8^,^9^, an effect that involved the inhibition of chronic inflammation, fibroblast proliferation induced by TGF-β signaling, fibroblast differentiation into myofibroblasts, and collagen synthesis. In the present study, we used two-dimensional electrophoresis (2-DE) to separate proteins expressed in rat lung tissues. The separated proteins were analyzed using matrix-assisted laser desorption ionization time-of-flight mass spectrometry (MALDI-TOF/TOF MS) and identified using the obtained spectra.

Based on the results of our proteomic profile analysis, we found that α-tubulin was down-regulated in lungs with silicosis-induced fibrosis. This down-regulation was reversed by Ac-SDKP treatment, suggesting that α-tubulin potentially has a role in silicosis development and progression. The acetylation of α-tubulin on lysine 40 (K 40) was one of the earliest tubulin post-translational modifications discovered to regulate microtubule inner proteins and intracellular transport. α-Tubulin is deacetylated by histone deacetylase family member 6 (HDAC6) and is acetylated by α-tubulin acetyl transferase (α-TAT1 or Mec-17)^10^. Acetylated α-tubulin (α-Ac-Tub) has been used as a marker of ciliated cells and exhibits an ability to clear mucus from the airway^11^. In this study, the specific expression and location of α-Ac-Tub in a rat model of silicosis were identified, issues that have rarely been addressed in previous studies. In addition, the microtubule system and actin cytoskeleton have been found to participate in the regulation of myofibroblast differentiation, tissue remodeling, and organ fibrosis^12^. In the present study, we explored 1) the differential protein profile related to rats with silicosis that were treated with Ac-SDKP; 2) the expression dynamics of α-Ac-Tub in the rat silicosis model; and 3) the differential expression of α-Ac-Tub regulated by HDAC6 and α-TAT1 in terms of the anti-silicosis effect of Ac-SDKP, particularly the inhibition of myofibroblast differentiation induced by Ang II.

## Results

### Comparative proteomic analysis of the Ac-SDKP effect on the silicosis rat model

**Figure 1:**
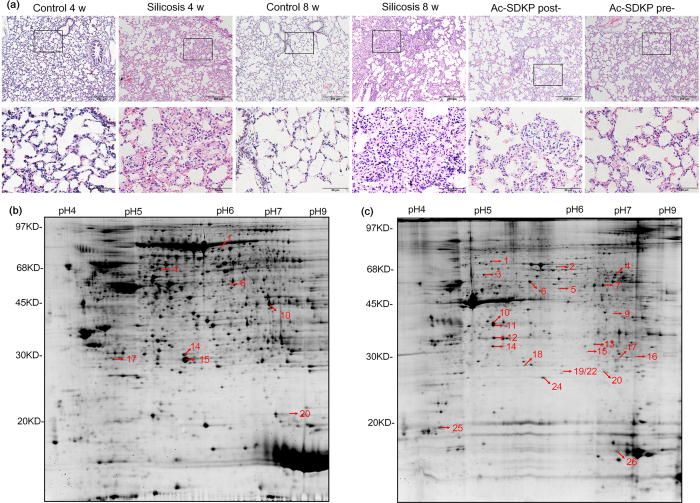
The pathological observation and 2-DE gel electrophoresis in rats. (**a**) Representative micrographs for H.E. staining in the lung from different groups as indicated. Scale bar = 200 μm and 50 μm. (**b**) Soluble protein extracts were analyzed in first dimension (pH 3–10 NL IPG, 24 cm); second dimension was performed on a vertical slab (13%T) gel. Protein detection was achieved by using colloidal Coomassie staining. (**c**) The 2-DE partern of insoluble protein extracts. Numbering refers to differentially-represented protein spots in the silicosis model, which were then excised, digested and identified by MS procedures as reported in Table S1–4.

H&E staining results of lungs obtained from rats are shown in Fig. 1a. In the disease model (4 w and 8 w) group, we observed silicosis nodules and interstitial fibrosis areas, and these pathological changes were alleviated in the Ac-SDKP post- and pre-treatment groups. Six samples from each group were analyzed using 2-DE profiling and MALDI-TOF-MS. In total, 2700 proteins (1400 soluble proteins and 1300 insoluble proteins) were identified in the 2-DE gel using ImageMaster 2D Platinum Software version 6.0 (Supplementary Table S1). As shown in Fig. 1, 2-DE patterns representative of rat lung tissues were displayed and distributed over an area of pI 4.0–8.0, with molecular weights of 14–97 kDa. Compared to the control group (4 w and 8 w), the silicosis model group (4 w and 8 w) and the Ac-SDKP treatment group (post- and pre-treatment) showed that seven soluble proteins (Fig. 1b) and 22 insoluble proteins (Fig. 1c) were differentially expressed. The peptide mass fingerprinting (PMF) of all 29 protein spots were successfully obtained, and their Mascot analysis results and differential expression patterns are listed in Supplementary Tables S1–4.

Gene ontology (GO) analysis was used to assess the GO distribution of the identified proteins. In the “molecular function” group, the identified proteins that functioned in binding (proteins, ions, GTP, ATP, enzymes and receptors) were ranked at the top of the category. In the “biological process” category, proteins that participated in cell proliferation and apoptosis, or that responded to stimuli, transport, metabolic and signaling pathways, constituted a high proportion of the identified proteins. The above data suggested that relevant functions were important in silicosis fibrosis and the anti-fibrotic effect of Ac-SDKP (Supplementary Table S5).

The protein expression patterns of lamin A (SB2) and HSP60 (SB3) in 2-DE gels were further validated using a Western blot analysis, and the results were similar to those observed in the 2-DE gels. Compared with the control group (4 w and 8 w), the expression of HSP60 increased while lamin A decreased in the silicosis model group (4 w and 8 w). These protein changes were reversed by Ac-SDKP treatment in both the post-treatment and pre-treatment groups compared with the silicosis 8 w group (Supplementary Figure S2 A). We also detected the expression of SB10 (ADRB-Gs protein complex) by immunofluorescence and Western blot. As showed in Supplementary Figure S1, the expression of Gs was decreased in the silicotic group.

### The expression profile of α-Ac-Tub in the silicotic rat and in fibroblasts induced by Ang II

**Figure 2:**
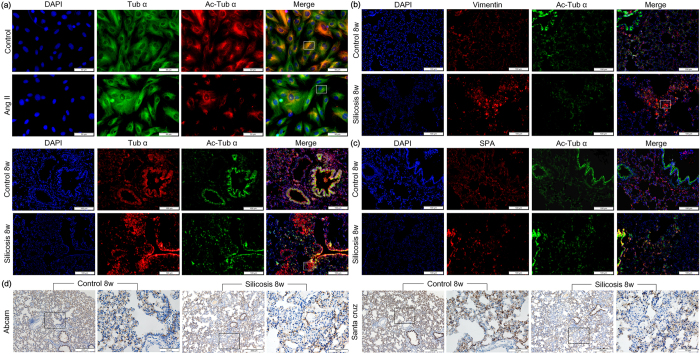
The co-expression of Tub-α and α-Ac-Tub in lung tissue and fibroblasts. (**a**) The co-expression of Tub-α and α-Ac-Tub in lungs of rat silicosis model and fibroblasts induced by Ang II measured by immunofluorescence. Scale bar = 100 μm and 50 μm. (**b**) The co-expression of α-Ac-Tub /vimentin in lung tissue. Scale bar = 100 μm. (**c**) The co-expression of α-Ac-Tub /SP-A in lung tissue. Scale bar = 100 μm. (**d**) The expression of α-Ac-Tub in silicosis assayed by immunohistochemistry with two different commercial antibodies. Scale bar = 200 μm and 50 μm.

Furthermore, we validated SA4 (α-Tub), another differentially expressed protein related to the anti-fibrotic effect of Ac-SDKP. Unexpectedly, the verification result was not consistent with the 2-DE result. Two different mechanisms can modulate microtubule diversity to adapt to a large variety of cellular functions. One mechanism is the presence of specific tubulin isotypes, and another is post-translational modifications (PTMs), among which acetylation had been previously identified as important for α-tubulin^10^. As shown in Fig. 2a, the expression of α-Ac-Tub was down-regulated in fibroblasts induced by Ang II for 24 h. Co-expression of α-Ac-Tub and α-Tub revealed by immunofluorescence showed that α-Ac-Tub exhibited specific expression in the alveolar walls of the lung tissue. Unlike the diffuse expression of α-Tub, a specific loss of α-Ac-Tub expression was observed in silicosis nodules.

Positive expression of α-Ac-Tub was previously demonstrated in fibroblasts^13^,^14^, epithelial cells^15^,^16^, and endothelial cells^17^,^18^ derived from lung tissue *in vitro*, and all these cell types have been suggested as potential sources of myofibroblasts^19^. To preliminarily determine which cell type positively expressed α-Ac-Tub, we examined the co-expression of α-Ac-Tub with the mesenchymal marker vimentin or with SP-A, a biomarker of alveolar type II cells, using immunofluorescence staining. As shown in Fig. 2b, positive co-expression of α-Ac-Tub with either SP-A or vimentin was observed in the alveolar walls of lung tissue, suggesting that the positive expression of α-Ac-Tub could be detected in alveolar type II and mesenchymal cells. In silicosis nodules, there was sporadic or weak expression of SP-A or α-Ac-Tub, and there was strong expression of vimentin, which was not accompanied by the co-expression of α-Ac-Tub. To eliminate the potential confounding effects of these antibodies, two different commercial antibodies were used, and similar expression patterns of α-Ac-Tub in rat lung tissue were obtained (Fig. 2c).

### Ac-SDKP stabilized the expression of α-Ac-Tub in a rat silicotic model

**Figure 3:**
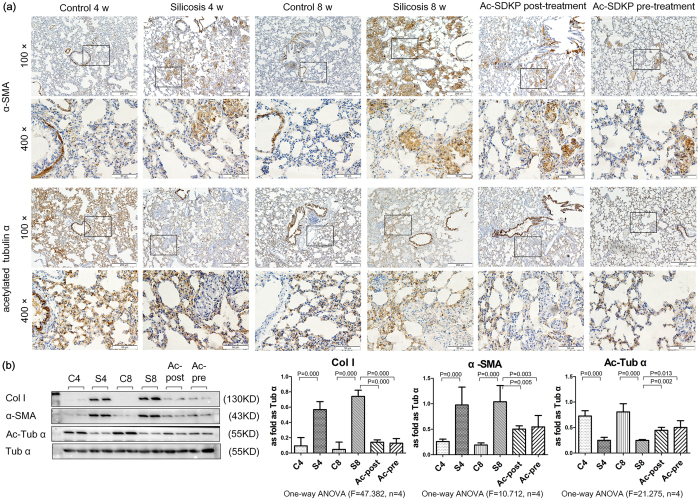
Effect of Ac-SDKP on col I, α-SMA, and α-Ac-Tub in silicosis rats. (**a**) The expression of α-SMA and α-Ac-Tub in lung tissue measured by immunohistochemistry. Scale bar = 200 μm and 50 μm. (**b**) The expression of col I, α-SMA and α-Ac-Tub in lung tissue measured by Western blot. Data presented as mean ± SEM; N = 4 independent experiments.

To better illustrate the role of interactions between microtubule acetylation and myofibroblast differentiation, we examined the location and expression of α-Ac-Tub and α-SMA in a rat model using IHC. As shown in Fig. 3a, and similar to our previous study^8^, positive staining for α-SMA was observed in vascular vessels and tracheal smooth muscle cells, but not in the interstitial space, in the control group. Specific expression of α-SMA was observed in silicosis nodules and interstitial fibrotic regions in the silicosis model. In contrast, in control animals that received saline treatment without SiO_2_ induction, the majority of detected α-Ac-Tub was expressed in the airway epithelium, similar to previous reports^11^; α-Ac-Tub also appeared in multiple cell types throughout the alveolar cells of the lung, as previously reported^20^. No positive expression of α-Ac-Tub was observed in silicosis nodules or interstitial fibrotic regions, even in the regions with positive α-SMA expression.

The immunostaining results were further confirmed using Western blot analysis (Fig. 3b), which showed that in the silicosis model (4 w and 8 w) group, silica induction increased the expression of α-SMA, which was accompanied by reduced expression of α-Ac-Tub compared with the control group (4 w and 8 w). Taken together, these results demonstrated that myofibroblast differentiation may result in microtubule disruption.

### Ac-SDKP attenuated loss expression of α-Ac-Tub in fibroblasts induce by Ang II

**Figure 4:**
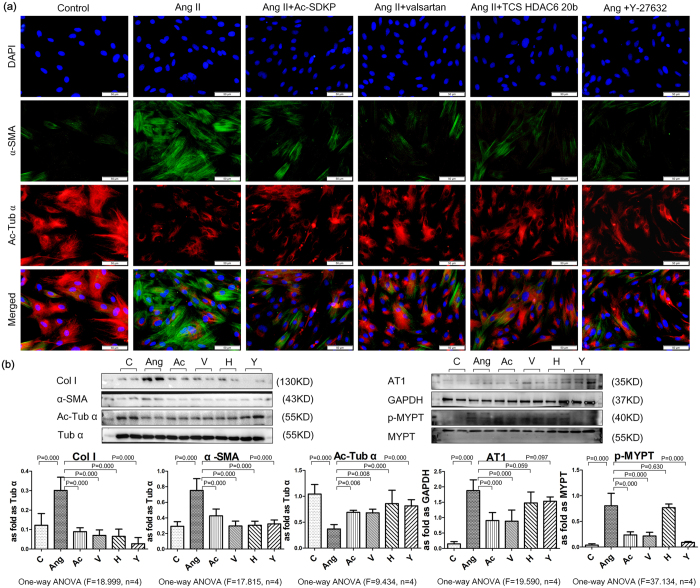
Effect of Ac-SDKP on col I, α-SMA and α-Ac-Tub in fibroblasts induced by Ang II. (**a**) The co-expression of α-SMA and α-Ac-Tub in fibroblasts induced by Ang II measured by immunofluorescence. Scale bar = 50 μm. (**b**) Effect of Ac-SDKP, valsartan (AT1 inhibitor), TCS HDAC6 20b (specific HDAC6 inhibitor), and Y-27632 (ROCK inhibitor) on fibroblasts measured by Western blot. Data presented as mean ± SEM; N = 4 independent experiments.

Similar to the effect of TGF-β1^8^, upon treatment with Ang II for 24 h, rat lung fibroblasts showed morphological changes, including cell widening and strong positive expression of α-SMA accompanied by decreased expression of α-Ac-Tub (Fig. 4). This finding suggested that the fibroblasts differentiated into a myofibroblast-like phenotype with microtubule disruption. Significantly up-regulated levels of α-SMA and col I protein, as well as down-regulated levels of α-Ac-Tub, were observed in response to Ang II treatment. To specifically examine the anti-fibrotic effect of Ac-SDKP, we synthesized mutant Ac-ADKP in which Ser was replaced by Ala, resulting in a non-functional analog of Ac-SDKP. As shown in Supplementary Figure S2, Ac-SDKP strongly inhibited the expression of col I and α-SMA, and it stabilized the expression of α-Ac-Tub induced by Ang II.

### Ac-SDKP inhibited the expression of HDAC6 *in vitro* and *in vivo*

Previous studies have suggested that microtubule deacetylation may be mainly regulated by HDAC6^10^. In the present study, we also found that the effects of Ang II on myofibroblast differentiation and ECM deposition may involve Ang II receptor type 1 (AT1), although there was no evidence of HDAC6 activation via AT1. To test the potential involvement of these mechanisms in the actions of HDAC6, we used pharmacological inhibitors of the corresponding receptors and enzymes. Treatment with valsartan (an AT1 inhibitor), TCS HDAC6 20b (a specific HDAC6 inhibitor^14^,^15^) and Y-27632 (a Rho-associated coiled coil-forming protein kinase (ROCK) inhibitor) caused α-Ac-Tub to be redistributed, and it attenuated the up-regulation of α-SMA and col I induced by Ang II. Treatment with Ac-SDKP showed similar results and inhibited the expression of AT1 and p-MYPT (Fig. 4).

**Figure 5:**
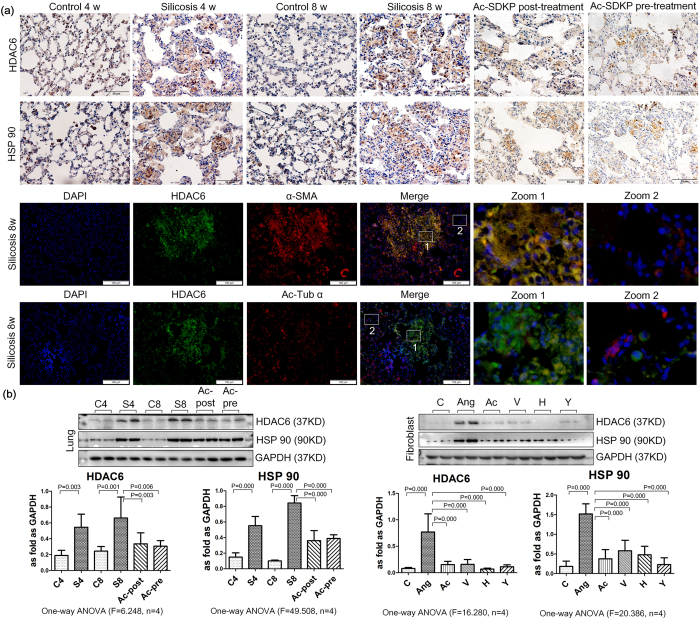
Effect of Ac-SDKP on expression of HDAC6 *in vivo* and *in vitro*. (**a**) The expression of HDAC6 and HSP 90 in lung tissue measured by immunohistochemistry. Scale bar = 50 μm. The co-expression of HDAC6/α-Ac-Tub and HDAC6/α-SMA measured by immunofluorescence in rat silicosis lung tissue. Scale bar = 100 μm. (**b**) Effect of Ac-SDKP on expression of HDAC6 and HSP 90 *in vivo* and *in vitro* measured by Western blot. Data presented as mean ± SEM; N = 4 independent experiments.

Furthermore, we observed the expression and localization of HDAC6 and HSP90 in rats with silicosis. As shown in Fig. 5a, in the rat lung, silicosis nodules had increased expression of HDAC6 and HSP90. *In vivo* and *in vitro*, the expression of HDAC6 and HSP90 was up-regulated in the silicosis group and in fibroblasts induced by Ang II. Upon post- or pre-treatment with Ac-SDKP, HDAC6 expression was decreased in rats. Moreover, treatment with Ac-SDKP, valsartan, TCS HDAC6 20b, and Y-27632 alleviated the elevated expression of HDAC6 induced by Ang II in fibroblasts (Fig. 5b).

### Ac-SDKP regulates the expression of α-TAT1 *in vitro* and *in vivo*

**Figure 6:**
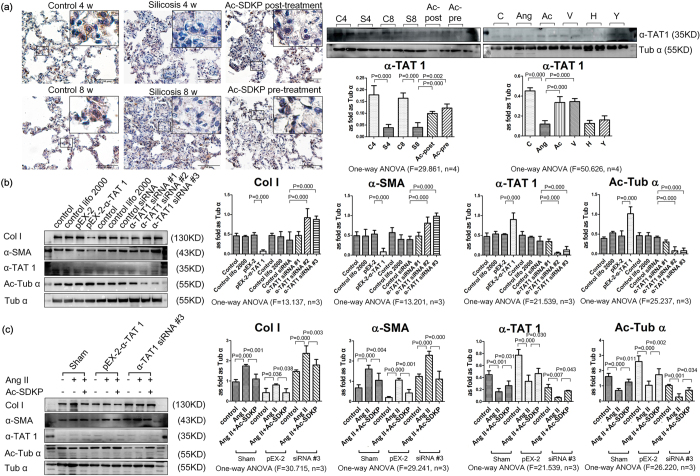
Ac-SDKP regulated the expression of α-TAT1 *in vitro* and *in vivo*. (**a**) The expression of α-TAT1 in lung tissue measured by immunohistochemistry. Scale bar = 50 μm. Effect of Ac-SDKP on expression of α-TAT1 *in vivo* and *in vitro* measured by Western blot. Data presented as mean ± SEM; N = 4 independent experiments. (**b**) Effect of over-expression or silence of α-TAT1 on col, α-SMA, α-TAT 1 and α-Ac-Tub in fibroblasts measured by Western blot. Data presented as mean ± SEM; N = 3 independent experiments. (**c**) Effect of over-expression or silence of α-TAT1 on col, α-SMA, α-TAT 1 and α-Ac-Tub in fibroblasts treatment with Ang II and Ac-SDKP measured by Western blot. Data presented as mean ± SEM; N = 3 independent experiments.

As shown in Fig. 6a, positive staining for α-TAT1 was observed in multiple cell types throughout the alveolar cells of the lung. No positive expression of α-TAT1 was observed in silicosis nodules or interstitial fibrotic regions, even in the regions with positive α-SMA expression. The immunostaining results were further confirmed using Western blot analysis, in which pre-treatment and post-treatment with Ac-SDKP reversed the reduced expression of α-TAT1 induced by silica. In addition, treatment with Ac-SDKP and valsartan reversed the reduced expression of α-TAT1 induced by Ang II in fibroblasts. Figure 6b showed that treatment of fibroblasts with α-TAT1siRNA to knockdown α-TAT1 resulted in a decrease of α-TAT1 and α-Ac-Tub, accompanied by increased expression of α-SMA and Col I. In contrast, over-expression of α-TAT1 resulted in an opposite effect. Furthermore, α-TAT1siRNA knockdown partially blocked the inhibitory effect of Ac-SDKP on Col I and α-SMA in fibroblasts induced by Ang II (Fig. 6c). As further shown in Fig. 6c, over-expression of α-TAT1 inhibited the increased expression of Col I and α-SMA induced by Ang II, and co-incubation with Ac-SDKP prolonged the inhibitory effect of pEX-2. These results suggested that over-expression of α-TAT1 was responsive to Ang II and Ac-SDKP.

## Discussion

Our group previously reported that Ac-SDKP has a beneficial effect upon silicosis, which involved the attenuation of TGF-β1 and Ang II signaling, pulmonary fibroblast proliferation, and collagen synthesis *via* c-Jun N-terminal kinase (JNK) signaling, as well as the regulation of myofibroblast differentiation by serum response factor (SRF)^8^,^9^. Other groups have also found that Ac-SDKP can inhibit fibrosis in organs including the heart, kidney, liver, and lung. Moreover, Ac-SDKP exerts multiple functions, such as anti-inflammation, anti-apoptosis, anti-fibrosis, and pro-angiogenesis; thus, it may be a candidate target molecule for novel anti-fibrotic drugs^21^. To enhance our ability in identifying potential biomarkers that are affected by Ac-SDKP in a rat silicosis model, we employed 2-DE/MS technologies to identify new biomarkers of silicosis and target proteins of Ac-SDKP. In the present study, we discovered 29 unique proteins whose expression was significantly altered in the silicosis rat model group or the Ac-SDKP treatment group, and whose function has not been previously explored in the organ fibrosis field, particularly in silicosis disease.

The results of the current study revealed a link between microtubule disruption to the formation and progression of silicosis. In fetal lung fibroblasts (FLFs), adult lung fibroblasts (ALFs), and IPF lung fibroblast (ILF) lines, treatment with TGF-β1 can induce the up-regulation of Col I, myofibroblast differentiation, and cell proliferation, and it can reduce the expression of α-Ac-Tub^18^. In lung tissue, α-Ac-Tub has been established as a marker of ciliated cells in the tracheal epithelium and was sloughed or diminished in repairing trachea after chlorine exposure^22^ and diacetyl instillation^17^. In this study, we found strong positive expression of α-Ac-Tub in airway epithelial cells. However, the positive expression was observed in cells of the alveolar wall. More interestingly, the loss of α-Ac-Tub expression was observed in silicosis nodules, which were dramatically occupied by myofibroblasts marked by α-SMA. In lung fibrosis, myofibroblasts can differentiate from fibroblasts, perivascular cells, fibrocytes, and cells derived from the epithelial-mesenchymal transition (EMT)^19^. Our study showed that α-Ac-Tub is co-expressed with alveolus superficial active substance (SPA, a marker of type II alveolar epithelial cells) and vimentin (the marker of mesenchymal cell). This finding suggests that α-Ac-Tub is widely expressed in alveolar epithelial cells, fibroblasts, and endothelial cells, and this expression is lost when the cells differentiate into myofibroblasts. In addition, microtubule polymerization has been identified to play a role in myofibroblast differentiation by controlling actin stress fiber/megakaryoblastic leukemia-1 (MKL1)/SRF signaling, but not Smad-dependent gene transcription^12^. In a previous study, we found that Ac-SDKP could attenuate the myofibroblast differentiation and collagen deposition induced by TGF-β1/SRF signaling^8^. In the current study, we found that Ac-SDKP counteracted the pro-fibrotic effects of Ang II by up-regulating α-Ac-Tub. These data provide a new mechanism underlying the anti-silicosis effect of Ac-SDKP and its interactive effect with the renin-angiotensin system (RAS).

Moreover, acetylation of α-Tub is a common post-translational modification, which can be inhibited by histone deacetylase family member 6 (HDAC6) and SIRT2^10^. It was demonstrated that HDAC6-dependent deacetylation of α-Tub in a human lung type II epithelial cell line (A549) exposed to TGF-β1 induced EMT *via* Smad signaling, and disparate responses to α-Ac-Tub were observed in primary cultures of human pulmonary arterial endothelial cells and fibroblasts stimulated with TGF-β1^23^. It has also been reported that ROCK increases the activity of HDAC6, followed by a decrease in α-Tub acetylation, and promotes cell proliferation and migration^24^,^25^. In the present study, we also observed that induction with Ang II resulted in the up-regulation of α-SMA, Col I, and HDAC6, as well as the down-regulation of α-Ac-Tub. Pre-treatment with Ac-SDKP, valsartan, TCS HDAC6 20b, and Y-27632 blocked myofibroblast differentiation, down-regulated the expression of Col I and HDAC6, and up-regulated α-Ac-Tub in cultured fibroblasts. Furthermore, as a classical substrate of HDAC6, HSP 90 was activated by deacetylation of HDAC6, and its inhibitor had an anti-fibrotic effect^26^,^27^,^28^. We also found that the expression of HDAC6 and HSP 90 were primarily observed in silicostic lesions and co-expressed with α-SMA. Furthermore, treatment with Ac-SDKP inhibited the up-regulation of HSP 90 in silicotic rats and in fibroblast induced by Ang II. These results suggest that Ac-SDKP has a potential role on HDAC6 in lung fibrosis through inhibiting the activity of HSP 90 and stabilization of the expression of α-Ac-Tub.

However, HDAC6 is not entirely selective for α-Ac-Tub and has deacetylase-independent functions. In contrast, acetyl transferase α-TAT1 specifically acetylates α-Tub on lysine 40^10^. In cultured *α-TAT1* −/− fibroblasts, elevated proliferation was related to deficiency of contact inhibition^29^. In the present study, we also found that that siRNA knockdown of α-TAT1 led to increased expression of α-SMA, while over-expression of α-TAT1 revealed an opposite effect. Meanwhile, α-TAT1siRNA knockdown also inhibited the anti-fibrotic effect of Ac-SDKP on myofibroblast differentiation, collagen synthesis and α-Ac-Tub stabilization. In conclusion, the results of our study indicate that Ac-SDKP regulates HDAC6 and α-TAT1 activity, which leads to a marked increase in α-Ac-Tub expression, blocks myofibroblast differentiation and collagen deposition.

## Methods

### Animals

Animal care and treatment were performed according to the established guidelines. The protocols were approved by the Animal Care Committee of North China University of Science and Technology (2013–038). Specific pathogen-free male Wistar rats (age 3 w; weight 180 ± 10 g) were purchased from Vital River Laboratory Animal Technology Co. Ltd. (SCXY 2009–0004, Beijing, China). The rats were housed in a temperature-controlled facility with a 12-h light/dark cycle and received food and water according to the guidelines established by the North China University of Science and Technology.

### Silicosis disease model

The experimental protocol and silicosis disease model were as previously described^8^. Briefly, the rats were anesthetized with isoflurane and received either silica solution (50 mg/rat, 1 ml) or 0.9% saline (vehicle) by trachea instillation. Ac-SDKP [800 μg/(kg d), Bachem AG company, USA] or control (0.9% saline) was given *via* a mini-osmotic pump (Model 2 ml 4, DURECT Co. Ltd, USA) implanted into the abdominal cavity. Sixty rats were divided into 6 groups (10 per group): 1) control 4 w (induced with 0.9% saline; then treated with 0.9% saline for 4 weeks); 2) silicosis model 4 w [silicosis induced with 50 mg SiO_2_ (s5631, Sigma-Aldrich, St. Louis, MO, USA) in rat trachea, then treated with 0.9% saline for 4 w]; 3) control 8 w (pre-induced with 0.9% saline 48 h before 0.9% saline induction, then treated with 0.9% saline for 8 w); 4) silicosis model 8 w (pre-treated with 0.9% saline 48 h before 50 mg SiO_2_ induction, then treated with 0.9% saline for 8 w); 5) Ac-SDKP post-treatment (induced with SiO_2_, treated with 0.9% saline for 4 w, and then treated with Ac-SDKP for an additional 4 w); and 6) Ac-SDKP pre-treatment (pre-treated with Ac-SDKP 48 h before induction with SiO_2_, then continued Ac-SDKP treatment for 8 w). At the end of the experiment, lung tissues were lavaged with 0.9% saline to remove blood before resection and were snap-frozen in liquid nitrogen for subsequent protein extraction.

### Sample preparation for two-dimensional gel electrophoresis (2-DE)

Frozen lung tissues (6 per group) were ground in liquid nitrogen and homogenized in deionized lysis buffer A containing 40 mM Tris, 1% w/v DTT, 1% v/v IPG buffer 3–10NL and cocktail protease inhibitor mix (04693116001, Roche), followed by homogenization. Sample homogenates were incubated on ice for 30 min and centrifuged at 40000 *g* for 30 min at 14 °C. The supernatants were supplied with 2 M thiourea and 7 M urea and labeled “soluble protein”.

The remaining precipitates were washed with lysis buffer A twice and re-suspended in lysis buffer B (2 M thiourea, 7 M urea, 40 mM Tris, 1% w/v DTT, 1% v/v IPG buffer 3–10 NL and cocktail protease inhibitor mixture). Next, the homogenates were centrifuged at 40,000 *g* for 30 min at 14 °C, and the supernatants were labeled “insoluble protein”.

### 2-DE image analysis, and protein identification (MALDI-TOF-MS)

A 2-DE protocol was followed as previously described^30^. Protein samples (1.3 mg, n = 6 per group) were loaded onto Immobiline dry strips (24 cm, pH 3–10 NL, GE Healthcare). Using IPGphor-3 (GE Healthcare), one-dimensional isoelectric focusing (IEF) was performed following the protocol provided by GE Healthcare. After equilibration in a buffer [6 M urea, 50 mM Tris base pH 8.8, 30% (v/v) glycerol, 2% (w/v) SDS (sodium dodecyl sulfate)] supplemented with 1% (w/v) DTT for 15 min, a second incubation was performed in the same buffer supplemented with 2.5% (w/v) iodoacetamide for an additional 15 min. Strips were then loaded onto 12% SDS polyacrylamide gels in an Ettan DALT II system (GE Healthcare). The proteins in the gel were visualized using Coomassie Blue G-250 (Amresco) staining and were scanned and analyzed using ImageMaster 2D Platinum Software version 6.0 (GE Healthcare). Only spots with at least a 1.5-fold change in vol% were considered differentially expressed.

Proteins with significant changes in expression were excised from the gels, washed, dehydrated, and digested with 10 ng/ml trypsin (Promega) in 50 mmol/L NH_4_HCO_3_. The peptide mixtures were deposited on a stainless steel MALDI probe and were dried slowly at ambient temperature. MS was performed using a Bruker Daltonics Autoflex MALDI-TOF-MS. Mass spectra were detected in the reflection mode and were recorded using FlexControl software (version 2.4, Bruker Daltonics) with the default parameters unless otherwise specified. Mono-isotopic peptide masses were labeled using Xmass software (version 5.1.1, Bruker Daltonics) with the default parameters unless otherwise specified.

Protein identification from the MS data was accomplished using the Mascot search engine (www.matrixscience.com/cgi/search_form.pl?FORMVER=2&SEARCH=PMF) with *Rattus* as the selected taxonomy and the NCBI non-redundant database selected for the search. Proteins were identified based on the following criteria^31^: (1) the score was greater than 60 (*p* < 0.05, default threshold); (2) the proportion of the theoretical protein sequence covered by MS data was greater than 20%; and (3) compared with theoretical values, the molecular mass variation was less than 30% and the pI variation was less than 2.0.

### Cell culture

Rat lung fibroblasts were isolated from 3-week-old Wistar rats using a trypsin digestion method and were maintained in complete culture medium [DMEM (BI-SH0019, BI, Kibbutz Beit-Haemek, Israel) supplemented with 10% fetal bovine serum (FBS, 10099141, Gibco, Thermo Fisher Scientific) and 100 U/ml penicillin/streptomycin] in a 37 °C incubator with 5% CO_2_. Cells at 80% confluence were cultured in FBS-free MEM medium for 24 hours, when most cells were in quiescent state. Next, the cells were divided into 6 groups and were cultured for 24 hours: 1) control; 2) 100 nmol/L Ang II (A9525, Sigma-Aldrich); 3) Ang II + 10^−8^ mol/L Ac-SDKP (H-1164, Bachem AG, Switzerland); 4) Ang II + 10^−6^ mol/L valsartan (V0112, Tokyo Chemical Industry, Tokyo, Japan); 5) Ang II + 10^−6^ mol/L TCS HDAC6 20b (4805, Tocris Bioscience, Bristol, United Kingdom); 6) Ang II + 10^−3^ mol/L Y-27632 (10005583 m, Cayman Chemical Company, Ann Arbor, MI, USA).

### Gene silencing and over-expression of *α-TAT 1*

Three siRNA against *α-TAT 1* were synthesized by Guangzhou Ruibo Biological Technology Co., LTD. The sequences of these siRNA were 1) Sense: 5′ GCAGCAAAUCAUGACUAUU dTdT 3′; Antisense: 3′ dTdT CGUCGUUUAGUA CUGAUAA 5′; 2) Sense: 5′ CCUCCAGUGACAGAGAAUU dTdT 3′; Antisense: 3′ dTdT GGAGGUCACUGUCUCUUAA 5′; 3) Sense: 5′ GGAACCUACGCAUACAG UU dTdT 3′; Antisense: 3′ dTdT CCUUGGAUGCGUAUGUCAA 5′. Control siRNA or *α-TAT 1* siRNA were transfected into cells using lipofectamine^®^ 2000 reagent (11668, Invitrogen, Carlsbad, CA, USA) according to the manufacturer’s instructions. Then, cells were incubated for 48 h before treatments with Ang II or Ac-SDKP were administered.

The coding sequence of *α-TAT 1* (1266 bp) was cloned into the *XhoI* and *BamHI* sites in transient expression vector pEX-2 by Shanghai Gemma Pharmaceutical Technology Co., LTD. Empty vector pEX-2 and pEX-2-α-TAT 1 were transfected into cells using lipofectamine^®^ 2000 reagent (11668, Invitrogen, Carlsbad, CA, USA) according to the manufacturer’s instructions. Then, cells were incubated for 48 h before treatments with Ang II or Ac-SDKP were administered.

### Immunocytochemistry (ICC) and immunohistochemistry (IHC)

Cells growing on chamber slides and paraffin-embedded tissue sections were used for ICC and IHC. Endogenous peroxidases were quenched with 3% H_2_O_2_, and antigen retrieval was performed using a high-pressure method on dewaxed tissue sections. The samples were then incubated with primary antibodies against α-SMA (ab3275, Eptomics, Mitten, CA, USA), α-Ac-Tub (sc-23950, Santa Cruz Biotechnology, USA or ab125356, Abcam, Cambridge, MA, USA), HDAC6 (20183, Arigo biolaboratories, Hsinchu, Taiwan, China) and HSP 90 (BD Transduction Laboratories, Chestnut Hill, MA, USA ) overnight at 4 °C, followed by incubation with a secondary antibody (PV-6000, Beijing Zhongshan Jinqiao Biotechnology Co. Ltd, China) at 37 °C for 20 min. Immunoreactivity was visualized with DAB (ZLI-9018, ZSGB-BIO, Beijing, China). Brown staining was considered a positive result.

For immunofluorescence staining, chamber slides or paraffin-embedded sections were blocked with 10% donkey serum (92590, Temecula, CA, USA) for 15 min. After co-incubation overnight at 4 °C with α-Ac-Tub/α-Tub (AF7010, Affinity Biosciences, Cincinnati, OH, USA), α-Ac-Tub/vimentin (ab92547, Abcam) and α-Ac-Tub/pulmonary surfactant-associated protein A (SPA, EL925092, Eterlife, Birmingham, UK), HDAC6/α-SMA and HDAC6/α-Ac-Tub the cells were incubated with Alexa Fluor^®^ 488 donkey anti-rabbit IgG (H + L) (A21206, Molecular Probes, Carlsbad, CA, USA) and Novex^®^ donkey anti-mouse TRITC affinity (A16016, Molecular Probes, Carlsbad, CA, USA), or rhodamine-labeled goat anti-rabbit IgG (H + L) (03-15-06, Kirkegard and Perry Laboratories, Gaithersburg, MD, USA) and fluorescein-labeled goat anti-mouse IgG (H + L) (02-18-06, Kirkegard and Perry Laboratories) for 60 min each at 37 °C in blocking buffer. Nuclei were stained with DAPI (C1002, Beyotime Biological Technology Co., LTD, Jiangsu, China). The cells or tissues were then visualized under an Olympus DP80 microscope and were analyzed using imaging software (cellSens 1.8, Olympus Corporation, Germany).

### Western blot analysis

The cells and lung tissue were lysed with RIPA buffer (BB-3201-1, BestBio, Shanghai, China), and the total protein content was quantified using the Bradford protein assay (PC0020, Solarbio; China). The proteins (20 μg/lane) were separated on a 10% gel by SDS-PAGE and were electro-transferred onto nitrocellulose membranes (Amervehicle Biosciences). The membranes were blocked with 5% non-fat milk and incubated overnight at 4 °C with a primary antibody [anti-collagen type I (ab34710, Abcam), anti-α-SMA; anti-α-Ac-Tub; anti-α-Tub; anti-AT1 (DF7592, Affinity Biosciences); anti-p-MYPT (AF5445, Affinity Biosciences); anti-MYPT (AF5444, Affinity Biosciences); anti-HSP 90; anti-HDAC6 and anti-α-TAT1 (ab58742, Abcam)], followed by a peroxidase-labeled affinity-purified antibody to rabbit/mouse IgG (H + L) (074–1506/074–1806, Kirkegard and Perry Laboratories). Target bands were visualized by the addition of ECL^TM^ Prime Western Blotting Detection Reagent (RPN2232, GE Healthcare, Hong Kong, China). The results were expressed as the fold change in α-Tub.

### Statistical analysis

Values were expressed as the means ± SEM. Comparisons between multiple independent groups were performed using one-way ANOVA followed by a post-hoc analysis with the Bonferroni test. Group differences with *p*-values less than 0.05 were considered statistically significant.

## Additional Information

**How to cite this article**: Xiaojun, W. *et al*. Acetylated α-Tubulin Regulated by N-Acetyl-Seryl-Aspartyl-Lysyl-Proline(Ac-SDKP) Exerts the Anti-fibrotic Effect in Rat Lung Fibrosis Induced by Silica. *Sci. Rep.* **6**, 32257; doi: 10.1038/srep32257 (2016).

## Supplementary Material

Supplementary Information
