# Supplementary material for: Acetylated α-Tubulin Regulated by N-Acetyl-Seryl-Aspartyl-Lysyl-Proline(Ac-SDKP) Exerts the Anti-fibrotic Effect in Rat Lung Fibrosis Induced by Silica
Source: Sci Rep. 2016 Aug 31;6:32257. doi: 10.1038/srep32257 (PMC5006047; doi:10.1038/srep32257)
Supplement: Supplementary Information [file srep32257-s1.pdf]

Acetylated α-Tubulin Regulated by N-Acetyl-Seryl-Aspartyl-Lysyl-Proline(Ac-SDKP) Exerts the Anti-fibrotic Effect in Rat Lung Fibrosis Induced by Silica

Wang Xiaojun[1#], Liu Yan[1#], Xu Hong[2#], Zhang Xianghong[1], Li Shifeng[2], Xu Dingjie[3], Gao Xuemin[2], Zhang Lijuan[2], Zhang Bonan[2], Wei Zhongqiu[2], Wang Ruimin[2], Darrell Brann[4], Yang Fang[1*]

1 Basic Medical Collage, Hebei Medical University, Shijiazhuang, China;

2 Medical Research Center, North China University of Science and Technology, Tangshan, China;

3 Traditional Chinese Medicine Collage, North China University of Science and Technology, Tangshan, China;

4 Department of Neuroscience and Regenerative Medicine, Medical College of Georgia, Augusta University, Augusta, GA 30912, USA.

[#] Equal contributors

* Correspondence: fangyang990404@sina.com

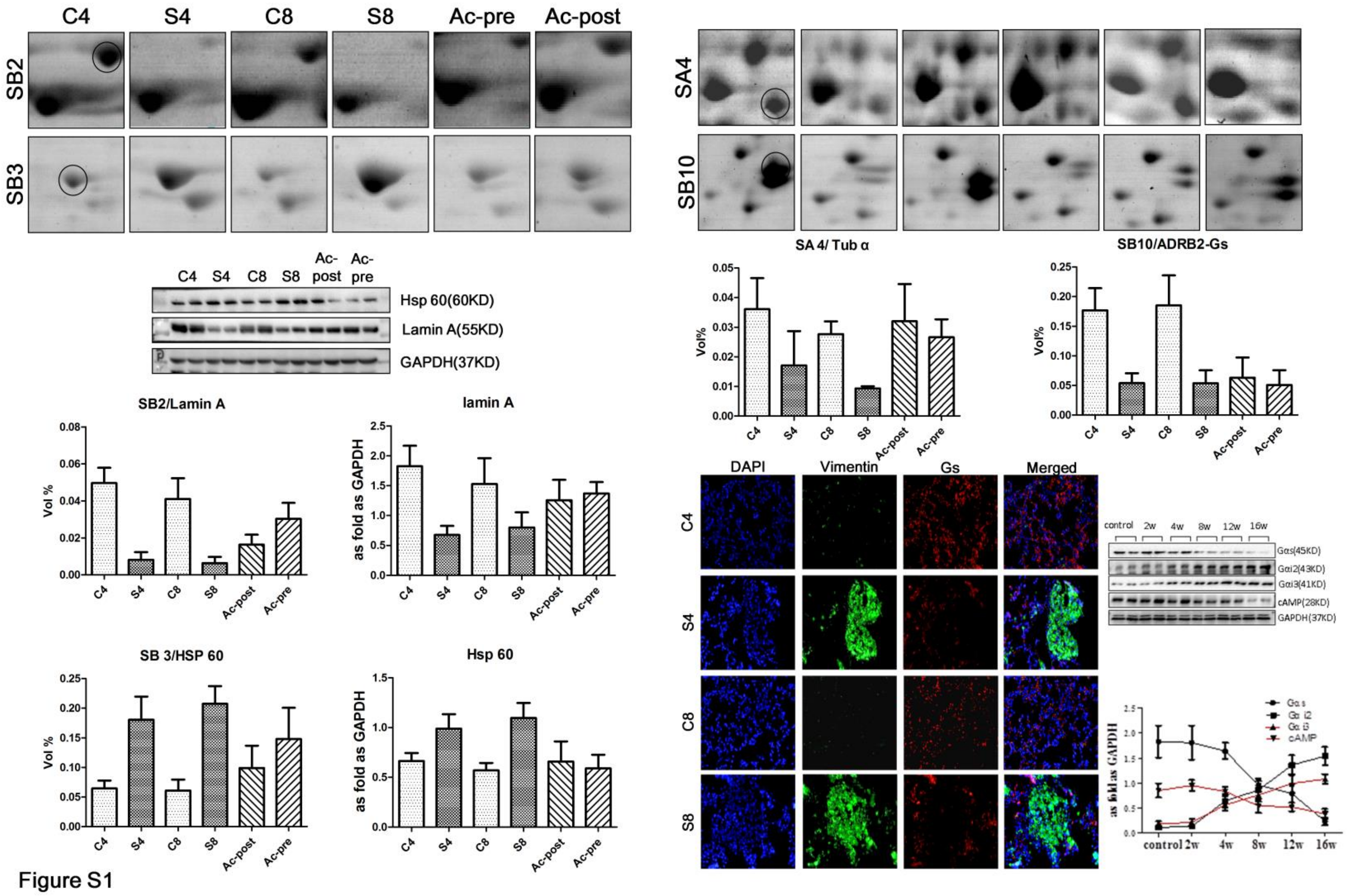

Figure S1 The differential protein validated by Western blot and immunofluorescence.

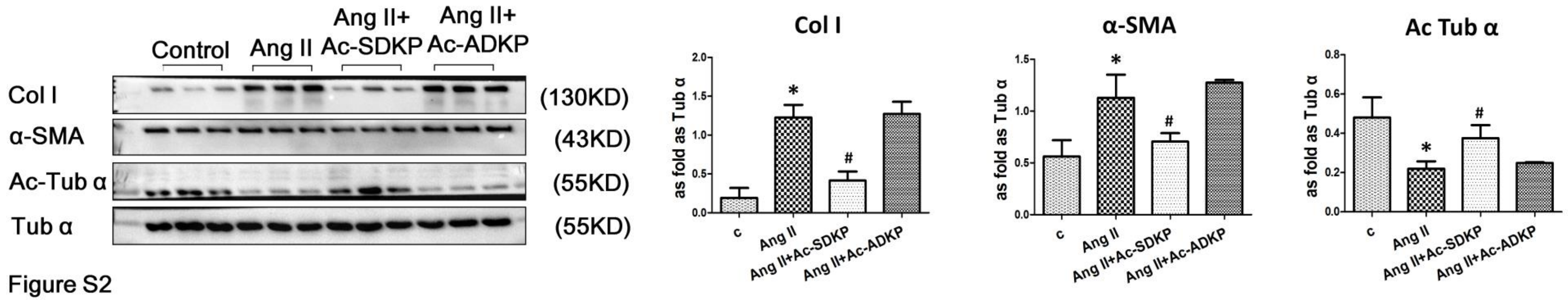

Figure S2 The specific effect of Ac-SDKP on Col I, α-SMA and Ac-Tub α in fibroblasts induced by Ang II

Table S1. Identification results of PMF of some differentially-expressed soluble proteins

No.	gi	Protein name	Gene name	PI (m)	MV(m) KD	PI(c)	MV(c) KD	Matched ratio	Seq cov	score
SA1	40254595	Dihydropyrimidinase-related protein 2 (DPYSL-2)	*Dpysl 2*	5.99	82	5.95	62	24/43	53%	199
SA4	34740335	tubulin alpha-1B chain	*Tuba 1b*	5.57	67	4.94	50	13/43	35%	120
SA6	1854476	Transferrin (TRF)	*Tf*	6.10	57	6.94	76	12/37	23%	64
SA10	158138555	aldo-keto reductase family, member C-like 1	*Akrlcl 1*	6.86	45	7.08	37	19/42	49%	152
SA14/15	94400790	heat shock protein beta-1 (Hsp 27)	*Hspb 1*	5.45	30	6.12	23	12/29	51%	157
SA17	57527565	rho GDP-dissociation inhibitor 2 (RhoGDIβ)	*Arhgdib*	5.13	28	4.99	23	5/13	35%	65
SA20	77680743	Ian2 protein	*Ian 2*	7.45	21	9.19	33	8/31	28%	70

Note: pI(m): measured pI value; MV(m): measured molecular weight; Seq cov: sequence coverage; pI(c):calculated pI value; MV(c): calculated molecular weight; Matched ratio: number of Mass values matched/searched

Table S2. Identification results of PMF of some differentially-expressed insoluble proteins

No.	gi	Protein name	Gene name	PI (m)	MV(m) KD	PI(c)	MV(c) KD	Matched ratio	Seq cov	score
SB1	1000439	75 KD glucose regulated protein (Hsp70)	*grp75*	5.45	88	5.87	75	20/28	32%	185
SB2	149048131	lamin A, isoform CRA_a	*Lmna*	6.00	86	6.37	65	21/33	36%	161
SB3	1334284	60 KD heat shock protein, mitochondrial(Hsp60)	*Hspd1*	5.35	76	5.91	60	20/46	39%	141
SB4	158186678	fibrinogen beta chain precursor	*Fgb*	6.96	77	7.90	54	22/46	44%	161
SB5	45737866	mitochondrial aldehyde dehydrogenase precursor (ALDH2)	*Aldh 2*	5.99	69	6.69	56	19/34	36%	178
SB6	203734	cytokeratin 8 polypeptide	*Krt8*	5.43	60	5.48	53	24/41	46%	182
SB7	38328220	Sept11 protein	*Sept 11*	6.59	69	6.36	50	12/20	26%	106
SB 9	149029697	rCG42519, isoform CRA_a	*rCG42519*	6.89	69	8.58	52	12/21	21%	104
SB10	340780680	Chain B, Crystal Structure Of The Beta2 Adrenergic Receptor-Gs Protein Complex	/	5.47	43	6.03	39	15/49	42%	81
SB11	4099537	G protein beta1 subunit	*rGb1*	5.48	43	5.47	37	13/46	63%	93
SB12	149069276	chloride intracellular channel 5, isoform CRA_b (CLIC5)	*Clic5*	5.50	38	6.33	24	12/24	57%	131
SB13	162719	ATP synthase alpha subunit precursor	*Atp5a1*	6.43	36	9.57	39	/	4%	155
SB14	6679299	Prohibitin (PHB)	*Phb*	5.48	34	5.57	30	16/46	60%	173
SB15	6755963	voltage-dependent anion-selective channel protein 1 (VDAC1)	*Vdac1*	6.37	34	8.62	31	9/24	40%	88

Table S2. continued

No.	gi	Protein name	Gene name	PI (m)	MV(m) KD	PI(c)	MV(c) KD	Matched ratio	Seq cov	score
SB16	302772633	hypothetical protein SELMODRAFT_92955	/	7.71	31	6.92	31	/	25%	82
SB17	114152094	Peptide-N(4)-(N-acetyl-beta-glucosaminyl) asparagine amidase (PNGase)	*nglyl*	7.12	31	6.20	75	10/18	17%	74
SB18	56587	ORF1	*ORF1*	5.80	29	9.51	41	8/36	24%	61
SB19/SB22	149029483	ATP synthase, H+ transporting, mitochondrial F1 complex, alpha subunit, isoform 1, isoform CRA_d	*Atp5a1*	6.06	28	8.24	55	19/33	34%	148
SB20	6729934	Chain A, Rat Liver F1-Atpase	*Atp5b*	6.67	28	8.28	55	21/38	30%	148
SB24	9506411	ATP synthase subunit d, mitochondrial	*Atp5h*	5.92	26	6.17	19	14/42	81%	158
SB25	159162414	Chain A, Structure Of The Hmg Box Motif In The B-Domain Of Hmg1	/	4.69	18	9.49	9	/	59%	156
SB26	34327944	myeloid-related protein-14 (MRP14)	*MRP14*	7.06	16	7.25	13	9/41	39%	66

Note: pI(m): measured pI value; MV(m): measured molecular weight; Seq cov: sequence coverage; pI(c):calculated pI value; MV(c): calculated molecular weight; Matched ratio: number of Mass values matched/searched

Table S3. Soluble protein (spot A) expression changes in different groups and shown as vol% (n=6)

No.	Protein name	Silicosis 4w *vs.* control 4w	P	Silicosis 8w *vs.* control 8w	P	Ac-SDKP anti-fibrotic *vs.* silicosis 8w	P	Ac-SDKP Pretreatment *vs.* silicosis 8w	P	F
SA1	Dihydropyrimidinase-related protein 2 (DPYSL-2)	↓ 25.22%	0.000	↓ 59.18%	0.022	↓ 50.00%	0.091	↑ 1.36-fold	0.219	9.022
SA4	tubulin alpha-1B chain	↓ 47.37%	0.001	↓ 33.57%	0.002	↑ 3.44-fold	0.000	↑ 2.86-fold	0.003	6.791
SA6	Transferrin (TRF)	↓ 51.31%	0.047	↓ 18.18%	0.002	↑ 9.41-fold	0.006	↑ 8.12-fold	0.067	3.684
SA10	aldo-keto reductase family, member C-like 1	↑1.46-fold	0.012	↑1.21-fold	0.198	↓50.41%	0.001	↓61.32%	0.006	6.148
SA14/SA15	heat shock protein beta-1 (Hsp 27)	↓ 47.42%	0.000	↓ 45.15%	0.007	↑ 1.38-fold	0.524	↑ 1.59-fold	0.213	5.940
SA17	rho GDP-dissociation inhibitor 2(RhoGDIβ)	↑ 3.38-fold	0.003	↑ 2.48-fold	0.002	↓ 71.85%	0.118	↓ 68.07%	0.078	5.012
SA20	Ian2 protein	↑ 2.58-fold	0.000	↑ 2.89-fold	0.001	↓ 62.12%	0.045	↓ 37.31%	0.000	9.369

Table S4. Insoluble protein (spot B) expression changes in different groups and shown as vol% (n=6)

No.	Protein name	Silicosis 4w *vs.* control 4w	P	Silicosis 8w *vs.* control 8w	P	Ac-SDKP anti-fibrotic *vs.* silicosis 8w	P	Ac-SDKP Pretreatment *vs.* silicosis 8w	P	F
SB1	75 KD glucose regulated protein	↑2.44-fold	0.004	↑2.81-fold	0.001	↓ 66.81%	0.068	↓ 84.77%	0.391	5.053
SB2	lamin A, isoform CRA_a	↓ 20.16%	0.000	↓ 15.37%	0.000	↑ 2.59-fold	0.026	↑ 4.81-fold	0.000	35.141
SB3	60 KD heat shock protein, mitochondrial	↑2.80-fold	0.000	↑3.40-fold	0.000	↓ 47.71%	0.000	↓ 71.37%	0.006	19.049
SB4	fibrinogen beta chain precursor	↓ 12.43%	0.000	↓ 23.06%	0.000	↑ 1.76-fold	0.140	↑ 2.07-fold	0.040	24.893
SB5	mitochondrial aldehyde dehydrogenase precursor	↑2.31-fold	0.004	↑2.99-fold	0.011	↓ 30.41%	0.066	↓ 45.50%	0.261	15.019
SB6	cytokeratin 8 polypeptide	↓ 35.73%	0.234	↓ 48.87%	0.004	↓ 10.22%	0.000	↓ 17.18%	0.000	12.887
SB7	Sept11 protein	↓ 40.32%	0.000	↓ 34.17%	0.000	↑ 1.13-fold	0.705	↑ 1.85-fold	0.040	15.723
SB 9	rCG42519, isoform CRA_a	↑4.47-fold	0.000	↑3.25-fold	0.001	↓93.94%	1.000	↓58.92%	0.026	37.177
SB10	Chain B, Crystal Structure Of The Beta2 Adrenergic Receptor-Gs Protein Complex	↓ 30.39%	0.000	↓ 28.98%	0.000	↑ 1.17-fold	0.626	↓ 94.41%	0.880	22.702
SB11	G protein beta1 subunit	↓ 29.93%	0.000	↓ 25.16%	0.000	↑ 1.38-fold	0.646	↑ 2.16-fold	0.025	16.228
SB12	chloride intracellular channel 5, isoform CRA_b	↓ 49.00%	0.000	↓ 47.96%	0.000	↑ 1.12-fold	0.379	↑ 1.76-fold	0.002	10.708
SB13	ATP synthase alpha subunit precursor	↑2.26-fold	0.000	↑2.56-fold	0.000	↓ 87.85%	0.228	↓ 63.99%	0.001	14.432

Table S4. continued

No.	Protein name	Silicosis 4w *vs.* control 4w	P	Silicosis 8w *vs.* control 8w	P	Ac-SDKP anti-fibrotic *vs.* silicosis 8w	P	Ac-SDKP Pretreatment *vs.* silicosis 8w	P	F
SB14	prohibitin	↑ 1.63-fold	0.000	↑ 1.74-fold	0.00	↓ 83.14%	0.054	↓ 82.78%	0.049	9.189
SB15	voltage-dependent anion-selective channel protein 1	↑ 3.23-fold	0.000	↑ 2.02-fold	0.000	↓ 99.31%	0.949	↓ 71.77%	0.003	23.472
SB16	hypothetical protein SELMODRAFT_92955	↑ 5.25-fold	0.000	↑ 2.07-fold	0.002	↓52.42%	0.003	↓ 72.97%	0.094	9.703
SB17	PNGase	↑ 1.92-fold	0.000	↑ 2.58-fold	0.000	↓ 65.91%	0.010	↓ 87.40%	0.319	8.736
SB18	ORF1	↑ 2.19-fold	0.000	↑ 2.01-fold	0.000	↓ 86.36%	0.064	↓ 80.68%	0.010	23.239
SB19/22	ATP synthase, H+ transporting, mitochondrial F1 complex, alpha subunit, isoform 1, isoform CRA_d	↑ 3.22-fold	0.000	↑ 2.78-fold	0.000	↑ 1.01-fold	1.000	↓64.77%	0.014	68.817
SB20	Chain A, Rat Liver F1-Atpase	↑ 3.21-fold	0.000	↑ 2.76-fold	0.002	↑ 1.02-fold	1.000	↓74.63%	0.574	23.466
SB24	ATP synthase subunit d, mitochondrial	↑ 1.91-fold	0.000	↑ 1.67-fold	0.001	↑ 1.02-fold	0.847	↓83.92%	0.144	8.924
SB25	Chain A, Structure Of The Hmg Box Motif In The B-Domain Of Hmg1	↓ 43.31%	0.022	↓ 25.52%	0.005	↑ 4.48-fold	0.001	↑ 4.81-fold	0.001	5.017
SB26	myeloid-related protein-14	↑ 3.51-fold	0.003	↑ 1.70-fold	0.132	↑ 3.29-fold	0.000	↑ 2.29-fold	0.000	35.613

Table S5 Gene Ontology of difference proteins

Molecular Function (25)	Gene
Binding (20)	Protein-lipid binding (Atp5b); cell surface binding (Fgb; Atp5b; Hspd1; Atp5a1); alcohol binding (ADRB2); amine binding (ADRB2); ion binding (ORF1; Calr; Mrp14; Tf; Sod2; Atp5b); hormone binding (Calr; Hspd1); peptide binding (Calr; Hspd1); oxygen dinding (Sod2); drug binding (ADRB2); carbohydrate binding (Calr); nucleic acid binding (Calr; Sod2); nucleoside binding (Atp5b; Hspd1; Grp75; Atp5a1); lipopolysaccharide binding (Hspd1); protein binding (Krt8; Sod2; Atp5b; Hspd1; Atp5a1; Vdac1; Hspb1; Dpysl2; Calr; ORF1; ADRB2; Gnb1; Aldh2; Fgb; Grp75); nucleotide binding (Tuba1b; Ian2; Sept11; Atp5b; Hspd1; Grp75; Atp5a1; Vdac1)
Catalytic activity (7)	Dpysl2; Tuba1b; Gnb1; Aldh2; Sod2; Atp5b; Atp5a1
Transporter activity (6)	Atp5h; Tf; Atp5b; Atp5a1; Vdac1;Clic5
Signal transducer activity (3)	Gnb1; ADRB2; Atp5b
Structural molecule activity (2)	Tubab; lmna
Transcription regulator activtity (2)	Calr; Phb
Enzyme regulator activity (1)	Arhgdib
Channel regulator activity (1)	ADRB2
Antioxidant activity (1)	Sod2

Table S5 continued

Biological progress	Gene
development	Dpsyl2; Calr; Lmna; Aldh2; Krt8; ADRB2; Atp5a1; Phb; Sod2; Tagln2; Hmg1
differentiation	Dpsyl2; Calr; Tf; Ian2; Hspd1; Krt8; ADRB2; Sod2
cell cycle	Calr; Sept
proliferation	Calr; Tf; ADRB2; Gnb1; Atp5a1; Phb; Sod2; Hmg1
apotosis	Tf; Hspb1; Lmna; Hspd1; Aldh2; Krt8; ADRB2; Gnb1; Phb; Vdac1; Sod2; Hmg1; MRP14
response	drug(Dpysl2; Calr; Akrlcl; Hspd1; Phb; Sod2; Hmg1); cocaine (Dpysl2; Hspd1); interleukin (Tuba1b; grp75; Fgb; Sod2; Hmg1); hypoxia (Tf; Lmna; Hspd1; ADRB2; Gnb1); estrogen (Akr1c1; Hspd1); hormonal (Aldh2; Phb); lipopolysaccharide (Aldh2; Sod2; Hmg1; MRP14); alcohol (Phb; Sod2; MRP14); others (Hspb1; Krt8; Clic5; Vdac1)
adhersion /motility/ migration	adhersion(Calr; Atp5b); motility(Tf; sept11); migration(Hspb1; Lmna; Fgb; ADRB2; Hmg1; MRP14)
signaling pathway	Calr; Tf; Hspb1; Fgb; ADRB2; Gnb1; Sod2; MRP14
transcription	Calr; Tf; ADRB2; Sod2
infammation	Hspb1; Hspd1; ADRB2; Hmg1; MRP14
transport	Dpysl2; Tf; grp75; ADRB2; Clic5; Atp5a1; Vdac1; Atp5b; Sod2; Atp5h
metabolic	Akr1c1; ADRB2; Clic5; Atp5a1; Sod2; Atp5h
DNA replication	Calr; Hmg1; Phb
cytoskeleton organization	Dpysl2; Calr; Tf; Lmna; Hmg1; MRP14
protein folding/ stabilization	Calr; grp75; Hspd1
endocytosis	Dpysl2; ADRB2; Atp5b; Calr
catabolism	Dpysl2; Aldh2; Atp5a1; nglyl; Atp5b; Atp5h
biosynthetic	Atp5a1 (ADP); Atp5b (ATP); MRP14 (interin)

Table S5 continued

cellular component (25)	Gene
Cytoplasm (21)	Fgb; Phb; Atp5a1; Atp5b; Lmna; Aldh2; Clic5; Sod2; Hspd1; Dpysl2; Grp75; Arhgdib; Mrp14; Tf; Sept11; Hspb1; ADRB2; Atp5h; Krt8; Vdac1; Calr
Membrane (19)	Fgb; Phb; Atp5a1; Atp5b; Clic5; Sod2; Hspd1; Gnb1; Mrp14; Sept11; Tf; Hspb1; ADRB2; Atp5h; Krt8; VDAC1; Tagln2; Calr; ORF1;
Mitochondrion (10)	Dpysl2; Grp75; Phb; Atp5a1; Atp5b; Aldh2; Atp5h; Vdac1; Sod2; Hspd1
Nucleus (8)	Phb; lmna; Mrp14; ADRB2; Hspb1; Calr; Tagln2; Hspd1
Cytoskeleton (8)	Dpysl2; Tuba1b; MRP14; Lmna; Sept11; Hspb1; Krt8; Clic5
cell surface (6)	Fgb; Grp75; Atp5b; Hspb1; Hspd1; Calr
extracellular region (5)	Fgb; MRP14; Tf; Hspd1; Calr
Golgi apparatus (3)	Clic5; Hspd1; Calr
Endosome (3)	Tf; ADRB2; Hspd1
Cytosol (3)	Dpysl2; Hspd1; Calr

Figure 1

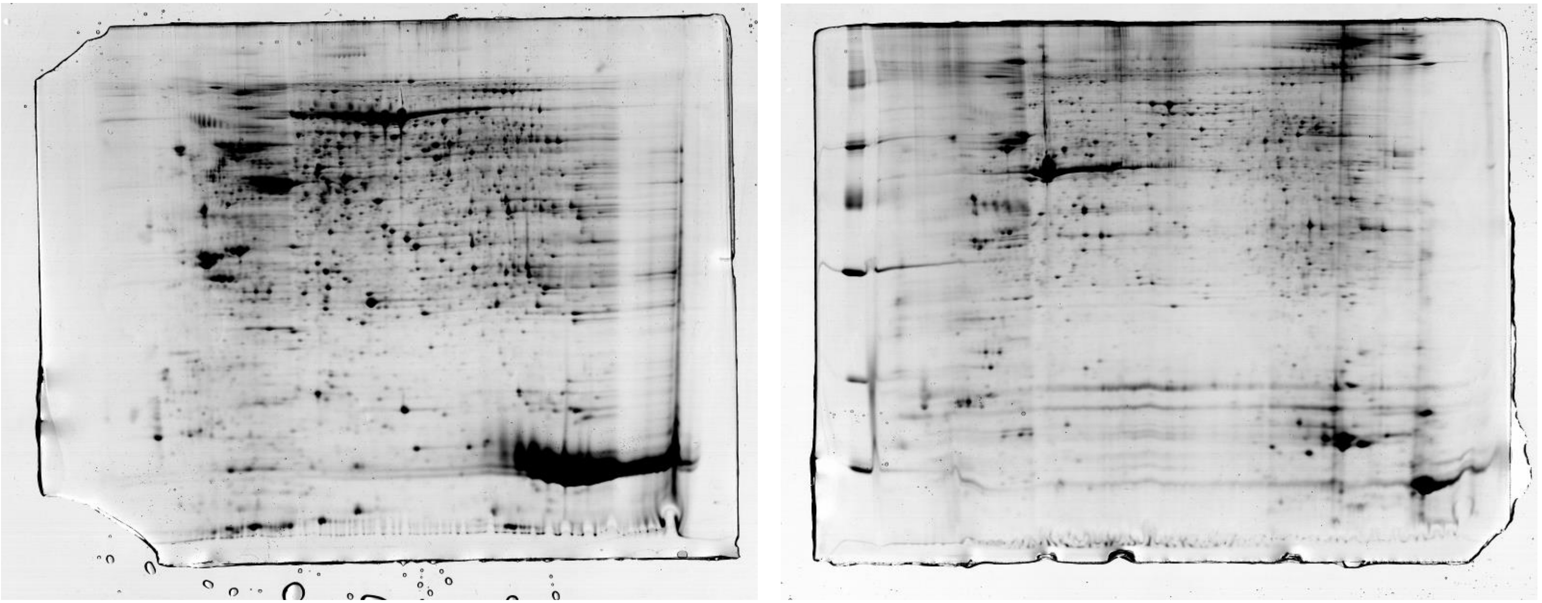

Figure 3

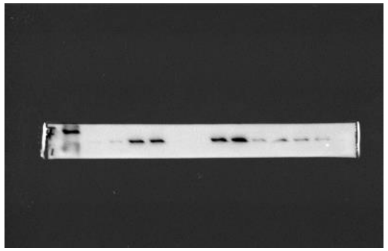

Col I

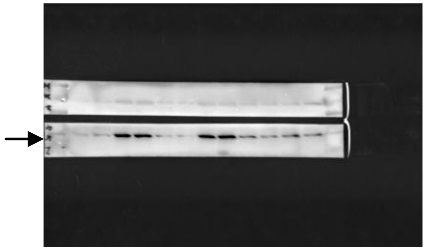

α-SMA

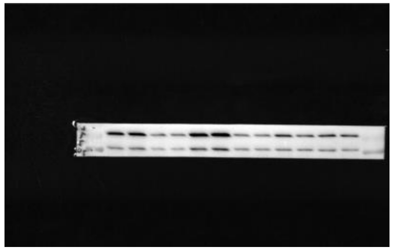

Ac-α-Tub

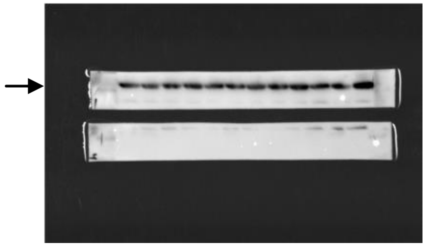

α-Tub

Figure 4

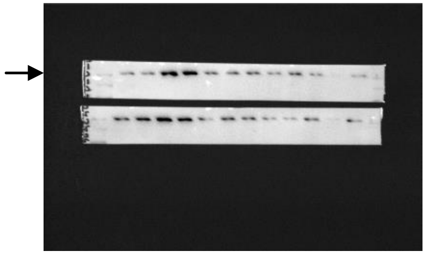

Col I

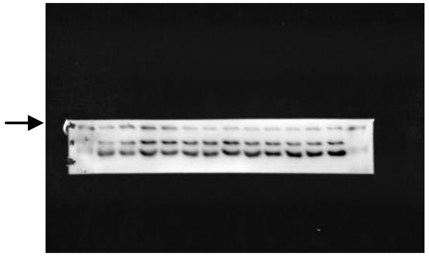

α-SMA

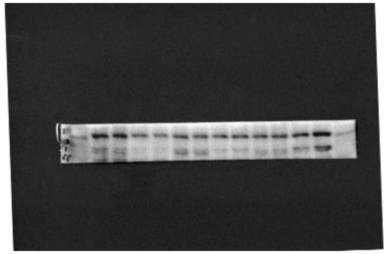

Ac-α-Tub

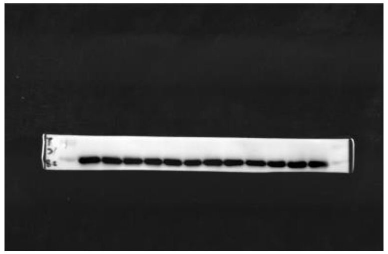

α-Tub

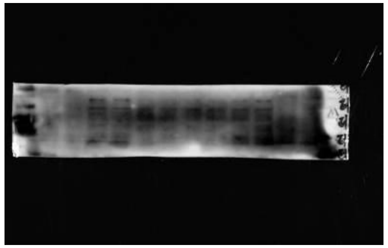

p-MYPT

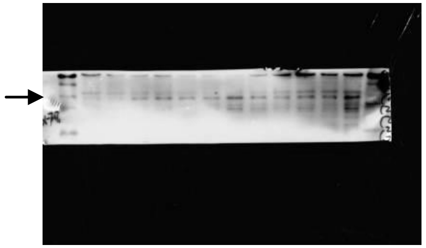

AT1

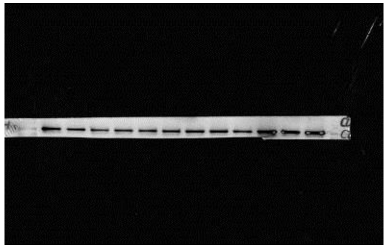

GAPDH

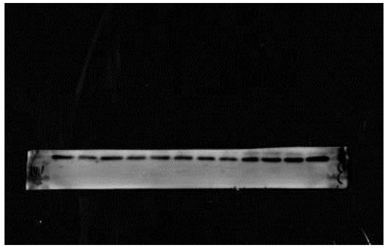

MYPT

Figure 5

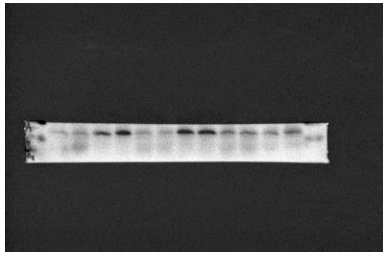

HDAC6

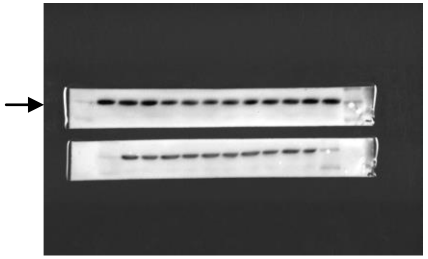

GAPDH

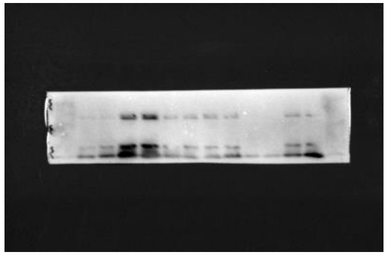

HDAC6

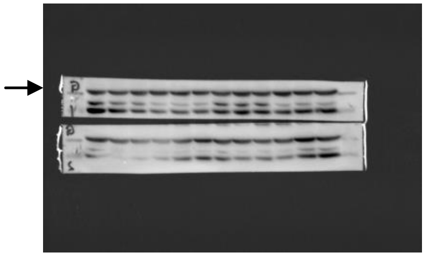

GAPDH

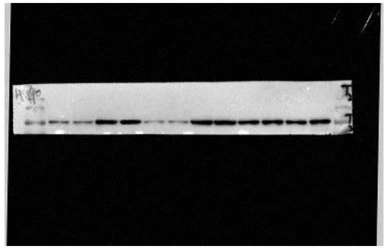

HSP 90

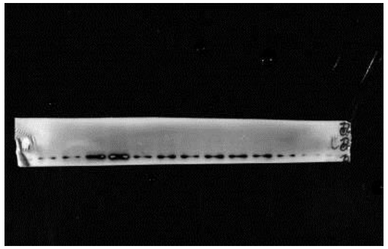

HSP 90

Figure 6

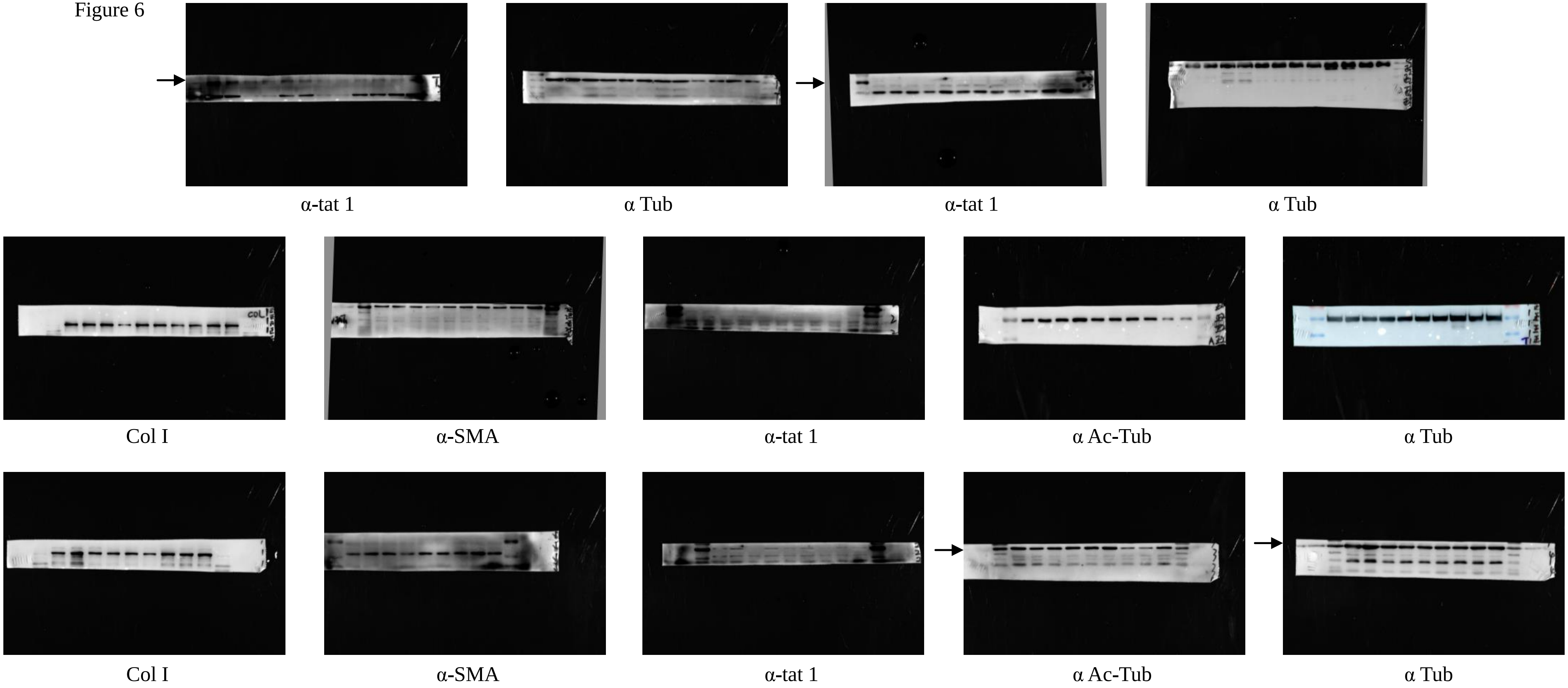
α-tat 1
α Tub
α-tat 1
α Tub
Col I
α-SMA
α-tat 1
α Ac-Tub
α Tub
Col I
α-SMA
α-tat 1
α Ac-Tub
α Tub
